# Cellular Signalling Networks in High Altitude Pulmonary Hypertension: From Canonical Pathways to Emerging Targets

**DOI:** 10.1111/cpr.70179

**Published:** 2026-02-10

**Authors:** Sheng Ding, Ju Chen, Zhaoyang Li, Yang Yu, Weijie Wang, Yan Liao, Jin Yang, Dianxiang Lu, Yujiang Fan

**Affiliations:** ^1^ Clinical Medical College & Affiliated Hospital, Chengdu University Chengdu P. R. China; ^2^ Medical College, Qinghai University Xining P. R. China; ^3^ College of Pharmacy, Chengdu University Chengdu P. R. China; ^4^ National Engineering Research Center for Biomaterials, Sichuan University Chengdu P. R. China

**Keywords:** cellular signal pathway, epigenetic regulation, high altitude pulmonary hypertension, intervention strategies

## Abstract

High altitude pulmonary hypertension (HAPH) is a complex disease featured by hypoxia‐induced pulmonary vasoconstriction and remodelling of small pulmonary arterioles, which could lead to increased pulmonary pressures and right ventricular hypertrophy and eventually result in heart failure. The temporal trajectory of HAPH progression can be divided into three overlapping phases: hypoxic pulmonary arterioles vasoconstriction, hypoxic pulmonary arterioles remodelling and even right heart failure. Each phase is governed by distinct molecular engines and cellular effectors that translate hypoxia physiological adaption into irreversible cardiopulmonary dysfunction. This review describes the intricate cellular signalling networks involved in the pathogenesis of HAPH, integrating canonical pathways such as HIF, MAPK and BMP with emerging targets like Wnt/β‐catenin, Notch, Hippo‐YAP and IL‐6. Inhibiting the HIF signalling pathway, modulating the MAPK pathway and suppressing the BMP, Wnt/β‐catenin, Notch, Hippo‐YAP and IL‐6 pathways have shown potential in reducing vascular remodelling and right ventricular dysfunction. Despite encouraging progress, the clinical translation remains constrained by a lack of deeper understanding of the signalling networks in HAPH. A comprehensive understanding of these signalling pathways in HAPH may yield critical insights into the disease's pathogenesis and facilitate the development of targeted intervention strategies. Future research should focus on elucidating the molecular mechanisms underlying these pathways, exploring genetic and environmental interactions, validating intervention targets, developing biomarkers, utilising systems biology approaches and conducting clinical trials.

## Graphical abstract

This review elucidates the intricate cellular signalling networks involved in high‐altitude pulmonary hypertension (HAPH), integrating canonical pathways like HIF, MAPK and BMP with emerging targets such as Wnt/β‐catenin, Notch, Hippo‐YAP and IL‐6. It highlights potential therapeutic strategies targeting these pathways to mitigate vascular remodelling and right ventricular dysfunction in HAPH.

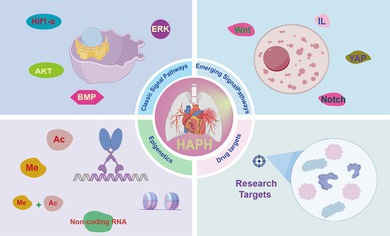

## 1 Introduction

Plateaus are natural laboratories of sustained hypobaric hypoxia. Above 2500 m, the nearly linear decline in barometric pressure reduces the inspired partial pressure of oxygen, elevating mean pulmonary artery pressure (mPAP) by about 3 mmHg for every 300 m gained [1]. This quantifiable pressure‐altitude relationship positions high altitude pulmonary hypertension (HAPH) as an environmentally induced form of pulmonary hypertension that uniquely allows the dissection of hypoxia‐driven vascular remodelling in genetically diverse human populations.

In contrast to acute high‐altitude illness, HAPH evolves insidiously: initial exertional dyspnea progresses, over months to years, to right‐ventricular hypertrophy and failure [2]. HAPH patients often have dyspnea, fatigue, chest pain and even syncope and their symptoms worsen with slight activity; their exercise capacity is severely limited. This not only forces patients to relocate from high altitude areas, but also leads to a significant decline in their ability to perform daily activities and seriously affects their quality of life. Pathologically, the disease is featured by hypoxia‐induced pulmonary arterioles vasoconstriction and hypoxic pulmonary arterioles remodelling of small pulmonary arteries—intimal thickening, medial hypertrophy and adventitial fibrosis—often without pronounced polycythemia, underscoring a primary role for the ‘hypoxia‐signal transduction‐vascular cell behaviour’ axis [3, 4, 5]. Epidemiologically, prevalence ranges from 5% to 35% in Andean, central‐Asian and Qinghai–Tibetan populations, whereas Tibetans with evolutionary adaptation exhibit lower incidence [6], highlighting gene–environment‐signalling network interactions as determinants of disease susceptibility.

Given that HAPH is initiated by a single, well‐defined environmental trigger, exhibits reversibility upon alleviation of hypoxia and progresses through clinically distinct stages, its underlying cellular signalling network provides a mechanistic paradigm for hypoxia‐associated pulmonary vascular diseases more broadly. In this review, we trace the temporal progression from acute hypoxia to chronic vascular remodelling and ultimately to right heart decompensation, integrating canonical signalling pathways (HIF, MAPK, BMP) with emerging regulators (Wnt/β‐catenin, Notch, Hippo‐YAP, IL‐6 classical and trans‐signalling). We delineate the spatiotemporal dynamics and cross‐talk among these pathways and discuss the potential strategies for facilitating precision interventions in future clinical and experimental contexts.

## 2 Epidemiology and Clinical Features of HAPH

**FIGURE 1:**
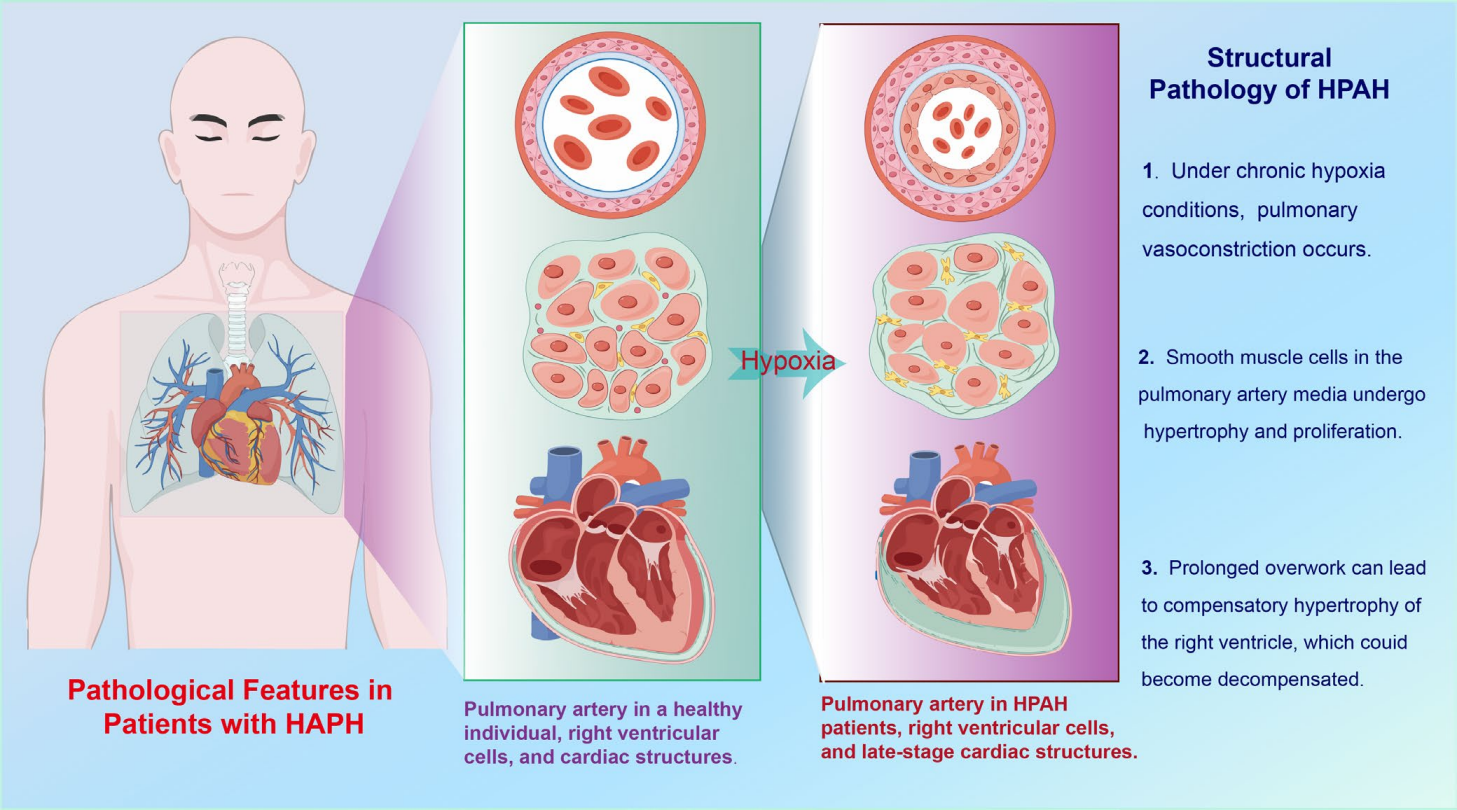
Background information on HAPH and International Classification Guidelines. Chronic hypoxia triggers pulmonary vasoconstriction and vascular remodelling, characterised by intimal hyperplasia and medial hypertrophy. These structural changes increase pulmonary vascular resistance and right ventricular afterload, potentially leading to right heart failure.

HAPH is characterised by a mPAP exceeding 30 mmHg or a systolic pulmonary artery pressure (sPAP) greater than 50 mmHg, confirmed through right heart catheterization [7, 8]. It occurs in individuals chronically exposed to altitudes above 2500 m, under hypoxic conditions, with other causes excluded. The primary pathological feature is hypoxia‐induced vasoconstriction and remodelling of small pulmonary arteries, typically without a significant increase in red blood cell count. In response to hypoxia, smooth muscle cells within the pulmonary arterioles proliferate and migrate, leading to vessel wall thickening and lumen narrowing, which in turn increases pulmonary vascular resistance and elevates pulmonary artery pressure. Furthermore, hypoxia impairs endothelial function by decreasing nitric oxide production and increasing endothelin‐1 levels, further exacerbating vascular remodelling and vasoconstriction. Histologically, affected individuals demonstrate intimal hyperplasia, medial hypertrophy and adventitial fibrosis within the pulmonary vasculature. These structural alterations progressively impair pulmonary circulation and increase right ventricular afterload, potentially resulting in right ventricular hypertrophy and failure [9, 10] (Figure 1).

HAPH initially presents with non‐specific symptoms, commonly including exertional breathlessness, fatigue and heart palpitations. As the condition advances, these symptoms exacerbate and patients may experience pronounced dyspnoea, chest pain and indications of right heart failure, including jugular venous distention, hepatomegaly and peripheral edema [11, 12]. These clinical manifestations substantially impair patients' quality of life and prognosis. Research indicates that individuals who have recently ascended to high altitudes or those who have not adequately acclimatised are at an elevated risk of developing HAPH. Globally, over 140 million people reside permanently at altitudes exceeding 2500 m, and more than 40 million individuals visit high‐altitude regions annually [13]. The prevalence of HAPH increases with altitude and is higher among males. Current research primarily focuses on populations in the Kyrgyz Plateau, Ethiopia, the Andes and China's Qinghai‐Tibet Plateau, with reported prevalence rates varying due to differences in diagnostic criteria (mPAP and study cohorts). In the Andes, prevalence ranges from 5% to 18%, whereas in Kyrgyz residents who fail to acclimatise, it may reach up to 14%. Among Central Asian populations living at altitudes around 3250 m, the prevalence ranges from 6% to 35%. In contrast, Tibetan populations, who have undergone long‐term adaptation to the Qinghai‐Tibet Plateau, exhibit a lower prevalence, ranging from 3% to 14% [14, 15, 16]. A study conducted in the Spiti Valley, at elevations between 3000 and 4200 m, reported a prevalence of 3.23% among permanent residents [15]. HAPH is considered reversible, with the potential for normalisation of mean pulmonary arterial pressure (mPAP) within 2 years after relocation to lower altitudes or following long‐term oxygen therapy [15]. Conventional vasodilators and targeted therapies are generally not recommended for HAPH, and advancements in pharmacological treatments have been limited, with few effective options currently available. Therefore, a systematic review of cellular mechanisms involved in HAPH pathogenesis may provide valuable insights for the identification and development of novel intervention agents.

## 3 Classical Signal Pathway

### 3.1 HIF Signal Pathway

Oxygen homeostasis is vital for cellular function and the hypoxia‐inducible factor (HIF) pathway plays a central role in cellular adaptation to low oxygen levels [17]. Hypoxia can arise under various physiological conditions, including high altitudes, exercise and embryonic development, as well as in pathological contexts like inflammation, tumour formation and lung disease. At high altitudes, the lower barometric pressure results in a decrease in arterial oxygen partial pressure, which impedes oxygen delivery to tissues [18]. The HIF family includes three subtypes: HIF‐1, HIF‐2 and HIF‐3, each playing distinct yet sometimes overlapping roles in the cellular response to hypoxia [19]. HIF‐1 is a heterodimer composed of HIF‐1α and HIF‐1β subunits, with HIF‐1α being rapidly induced in response to hypoxia. This subunit is involved in a wide range of cellular adaptations. For instance, in stromal cells of giant cell tumours of bone, HIF‐1α upregulates microRNA‐210, reflecting an adaptive response to hypoxia [20]. In haematopoietic stem cells (HSCs), in vitro studies indicate that PDK1, regulated by HIF‐1α, plays a crucial role in maintaining transplantable HSC function, even though the in vivo relationship between HIF‐1α and pyruvate dehydrogenase kinase 1 (Pdk1) remains unclear [21]. Known as endothelial PAS domain‐containing protein 1 (EPAS1), HIF‐2 is another key transcription factor that is responsive to low oxygen environments. In renal erythropoietin‐producing cells, HIF‐2α regulates erythropoietin (Epo) production [22]. HIF‐2α also plays a role in HAPH, contributing to vascular remodelling and vasoconstriction [23, 24, 25]. HIF‐3, in contrast, has a more complex and less well‐understood function. Some studies suggest that HIF‐3α may act as a negative regulator of the HIF pathway. It is suggested that alternative splicing of HIF‐3α produces inhibitory proteins that can compete with HIF‐1α and HIF‐2α for binding to HIF‐1β and DNA, thereby modulating transcriptional activity [26]. However, further research is needed to clarify the precise roles and regulatory mechanisms of HIF‐3 in various cell types and physiological/pathological conditions.

### 3.2 HIF Promotes Endothelial Apoptosis

**FIGURE 2:**
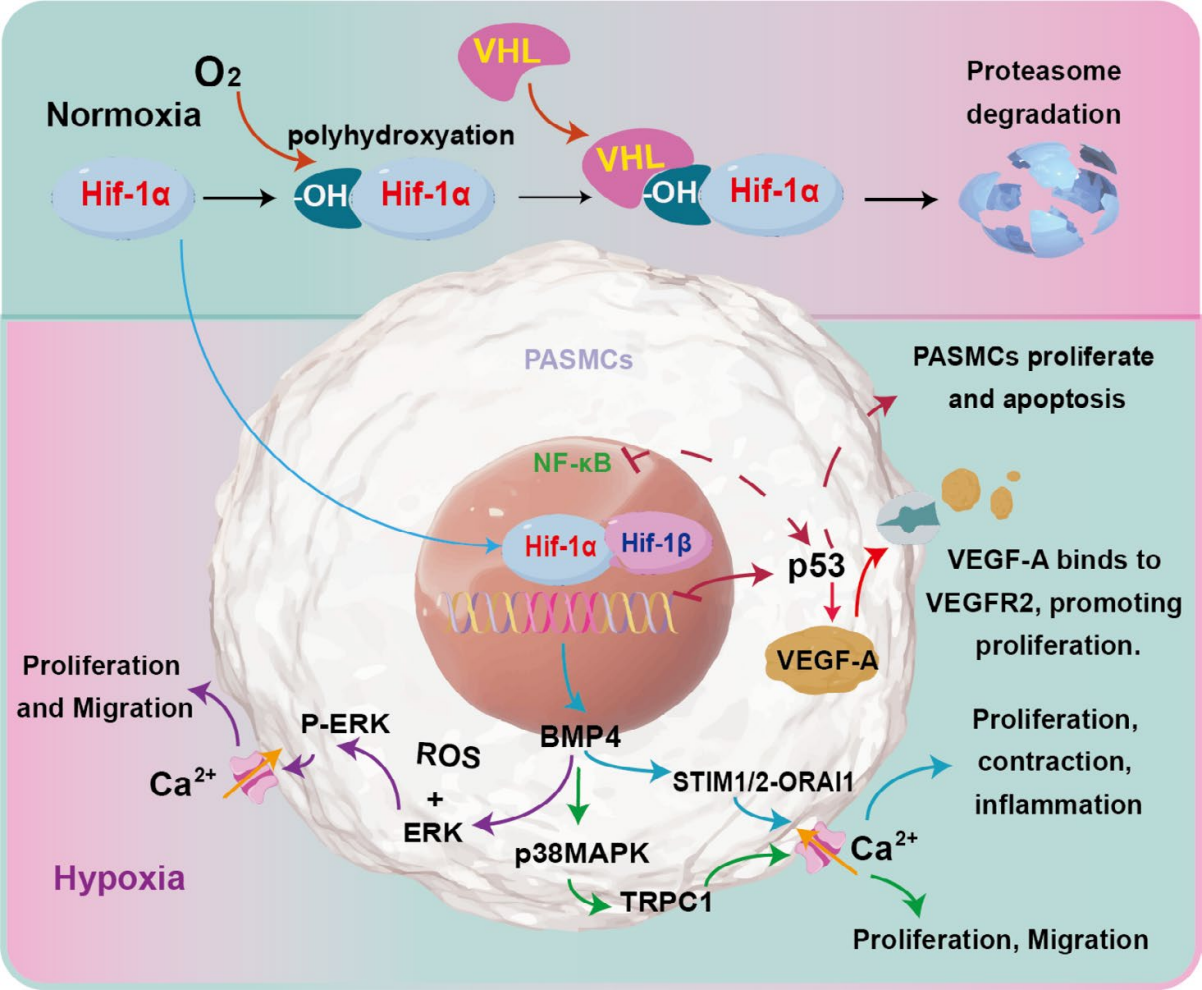
The central role of the hypoxia‐inducible factor (HIF) pathway in oxygen homeostasis. Under hypoxic conditions, the HIF‐α subunit is stabilised and dimerizes with HIF‐1β to form the active transcription factor. The primary HIF isoforms, HIF‐1 and HIF‐2, regulate distinct sets of target genes to coordinate cellular adaptation, including metabolic reprogramming and erythropoiesis. This response is critical for survival in low‐oxygen environments but can also contribute to pathological processes of HAPH.

Hypoxia is a significant factor in the pathogenesis of HAPH, where it upregulates HIF‐1α and HIF‐2α in pulmonary artery endothelial cells (PAECs), thereby promoting apoptosis [27, 28]. In a study on monocrotaline‐induced pulmonary arterial hypertension in rats, Astragaloside IV (ASIV) was found to reduce disease symptoms by improving inflammation, PAEC dysfunction and apoptosis [27]. Hypoxia‐induced HIF‐1α upregulation in human pulmonary artery smooth muscle cells (HPASMCs) was linked to increased proliferation and resistance to apoptosis (Figure 2), while ASIV treatment reversed these effects. Iron metabolism disturbances also play a role in HAPH, regulated by bone morphogenetic protein (BMP) signalling. In rats exposed to hypoxia for 4 weeks, there was an increase in right ventricular systolic pressure and right ventricle hypertrophy index, alongside a decrease in serum iron levels, reflecting iron metabolism disorders [28]. Iron supplementation reversed hypoxia‐induced oxidative stress and apoptosis in cultured human PAECs [29].

### 3.3 HIF Induces Vascular Changes

High‐altitude hypobaric hypoxia can lead to significant vascular changes in the lungs, with the HIF pathway implicated in these alterations. Hypobaric hypoxia reduces arterial oxygen saturation, inducing hypoxia in lung tissues and contributing to pulmonary complications, particularly in pulmonary arterioles, which underlie the pathophysiology of HAPH [30]. Both adaptive and maladaptive responses to hypoxia, including changes in oxygen sensing, hypoxia signalling and ion channels, are regulated by the HIF pathway. In a study on HAPH patients, NLRC3 deficiency was shown to promote HAPH via the IKK/NF‐κB p65/HIF‐1α pathway [31]. The use of immunohistochemistry revealed a reduction in NLRC3, which was co‐located with smooth muscle cells in the pulmonary vessels of HAPH patients. In mice, NLRC3 knockout led to more severe right ventricular hypertrophy and fibrosis in hypoxia‐induced models.

### 3.4 HIF Promotes Mitochondrial Dysfunction and Metabolic Reprogramming

Mitochondrial dysfunction and metabolic reprogramming are crucial features in the pathogenesis of HAPH, with the HIF pathway playing an integral role in these processes [32, 33]. In high‐altitude pulmonary oedema (HAPE), mitochondrial DNA mutations have been associated with increased oxidative stress and metabolic reprogramming during hypobaric hypoxia. Sequencing of mtDNA from HAPE subjects revealed mutations in Complex I genes, which affect protein structure and contribute to mitochondrial dysfunction [32]. Given that HAPE serves as a precursor to HAPH, mitochondrial dysfunction and metabolic reprogramming are likely to play significant roles in the pathogenesis of HAPH.

### 3.5 HIF Regulates Vasoconstriction

The HIF pathway is involved in regulating vasoconstriction in HAPH, with several mechanisms contributing to this process. One of the key mechanisms by which the HIF pathway influences vasoconstriction in HAPH is through the regulation of thrombospondin‐1 (TSP1) [23]. Furthermore, the HIF‐2α‐arginase axis has been identified as essential for the development of pulmonary hypertension, with endothelial HIF‐2α deletion protecting against hypoxia‐induced pulmonary vascular remodelling and hypertension [24]. The regulation of calcium sensitivity and reactive oxygen species (ROS) production are additional mechanisms through which the HIF pathway influences vasoconstriction. Chronic hypoxia increases ROS, enhancing pulmonary arterial constriction through calcium influx and myofilament sensitization. This process is mediated by HIF‐1α, which promotes adverse pulmonary vascular remodelling and hypertension [34, 35].

### 3.6 HIF Promotes Endothelial‐to‐Mesenchymal Transition (EndMT)

EndMT plays a significant role in HAPH pathogenesis and the HIF pathway has been shown to promote this process. In a study of intermittent hypoxia (IH), hsa_circ_0081065 exacerbated IH‐induced EndMT through regulation of the miR‐665/HIF‐1α axis and HIF‐1α nuclear translocation [36]. In HAPH, the upregulation of versican (VCAN) through promoter hypomethylation promotes EndMT [37].

### 3.7 HIF Promotes Cell Proliferation

The increase in HIF‐1α due to low oxygen levels is crucial for the growth of pulmonary artery smooth muscle cells in HAPH [38, 39]. In adult rats subjected to low oxygen levels, the introduction of HIF‐1α shRNA via lentivirus reduced the increase in HIF‐1α caused by hypoxia, easing pulmonary hypertension symptoms [38].

### 3.8 HIF Promotes ECM Remodelling

HIF‐1α‐induced upregulation significantly affects the angiogenic potential of PAECs and the migration of PASMCs and pulmonary arterial fibroblasts (PAFs) [40, 41]. In PAFs, hypoxia induces proliferation, migration and differentiation via the PI3K/Akt/p70S6K signalling pathway [41]. In mice with the R200W Vhl mutation, increased HIF‐2α levels promote ECM remodelling through the activation of myofibroblasts [42].

## 4 MAPK Signal Pathway

**FIGURE 3:**
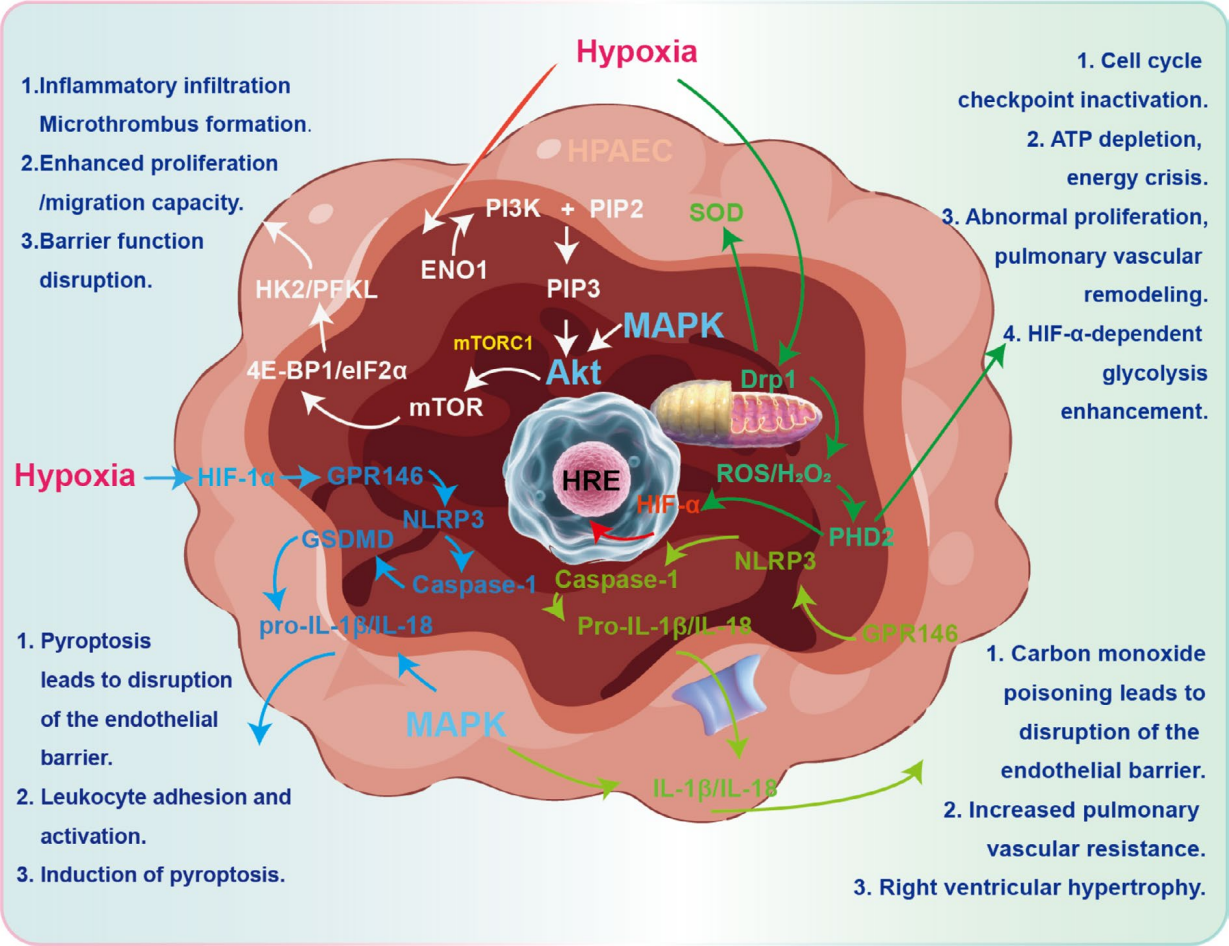
Schematic of the Ras‐Raf‐MEK‐ERK/MAPK signalling cascade and its functional outcomes. This cascade is a fundamental intracellular signalling module that transmits extracellular signals from activated receptors to the nucleus. Upon stimulation by growth factors or other cues, the sequential activation of mTOR and MAPK kinases occurs. The resulting phosphorylated MAPK translocates to regulate diverse cellular processes, including proliferation and survival.

The Ras‐Raf‐MEK‐MAPK (mitogen‐activated protein kinase) signalling pathway is a critical regulator of numerous cellular functions, including cell growth, differentiation and survival [43]. Initially identified in the early 1980s when Raf family kinases (A‐Raf, B‐Raf and C‐Raf) were recognised as retroviral oncogenes, the pathway has been extensively studied in relation to cancer, especially following the discovery of activating mutations in B‐Raf in various tumours [44]. MAPK, a serine–threonine protein kinase, serves as a central transducer, relaying signals from membrane receptors to various cytoplasmic and nuclear targets [45]. The pathway is activated by various stimuli, such as cytokines, growth factors, neurotransmitters, hormones, cell stress and cell adhesion [46] (Figure 3). For example, while genetic alterations in MAPK components are not a common mechanism in adrenocortical tumorigenesis, the pathway is often deregulated [47]. In melanoma, the MAPK pathway has been shown to promote tumour growth by modulating downstream signalling pathways like AKT/MAPK and NF‐κB/iNOS [48].

### 4.1 MAPK and Mitochondrial Dysfunction

Oxidative stress‐induced activation of p38 MAPK has been implicated in the development of HAPH, mainly by increasing ROS levels. Research on pulmonary hypertension revealed that leukotriene B4 (LTB4) promotes the growth, movement and specialisation of human pulmonary artery adventitial fibroblasts in a dose‐dependent way by triggering the p38 MAPK pathway [49]. This activation was associated with the upregulation of Nox4 and ROS production, which were essential for fibroblast activation and the progression of pulmonary hypertension. Inhibition of this pathway resulted in reduced inflammation, lower ROS production and decreased fibroblast activation, suggesting the p38‐Nox4 axis is crucial in the development of HAPH. In the context of pulmonary vascular remodelling under hypoxia, exogenous 15‐HETE facilitated ROS production, primarily in mitochondria [50]. The mitochondrial electron transport chain and Nox4 were found to contribute significantly to the 15‐HETE‐induced ROS increase, which in turn stimulated endothelial cell migration and PASMCs proliferation via the p38 MAPK pathway [50]. These findings suggest that ROS regulated by 15‐HETE and the p38 MAPK pathway may contribute to HAPH development through cell proliferation and migration. In a rat model of acute hypobaric hypoxia and exercise‐induced pulmonary oedema, Solnatide was shown to reduce pulmonary oedema and enhance gas–blood barrier function by improving occludin expression [51]. This study also highlighted the role of oxidative stress and the activation of pathways like p38 MAPK in the development of pulmonary edema, further supporting the importance of p38 in HAPH development.

### 4.2 MAPK and ECM Remodelling

In HAPH, the activated MAPK pathway plays a significant role in extracellular matrix (ECM) remodelling within the pulmonary vasculature and the right ventricle. In a study on right ventricular fibrosis, increased mitochondrial fragmentation mediated by Drp1 promoted fibroblast proliferation and collagen production [52]. Inhibiting Drp1 using mitochondrial division inhibitor 1 (Mdivi‐1) reduced mitochondrial fragmentation and collagen expression in right ventricle fibroblasts, suggesting that the MAPK pathway, possibly through its influence on mitochondrial dynamics, regulates ECM remodelling in HAPH. The role of MAPK in ECM remodelling was further supported by a study on MEKK2, a MAPK kinase, in HAPH. In MEKK2 knockout mice, the development of right ventricular hypertrophy was attenuated compared to wild‐type mice, highlighting the role of MEKK2 in regulating inflammation and ECM remodelling through the ERK5 pathway during HAPH [53]. ERK also contributes to ECM remodelling in HAPH. In a study on fibroblast collagen fibrillogenesis, Pten knockout increased collagen fibre production, providing insights into how ERK‐related pathways influence collagen synthesis, potentially relevant to ECM remodelling in HAPH [54]. Additionally, in cardiac fibrosis, WISP‐1 facilitated collagen processing in cardiac fibroblasts via the Akt and ADAMTS‐2 pathways, with ERK likely involved in this process [55]. This suggests that ERK‐mediated ECM remodelling is also relevant in fibrotic diseases like HAPH.

### 4.3 MAPK and Inflammation

The MAPK pathway has a significant role in promoting inflammation of HAPH. In human umbilical vein endothelial cells (HUVECs), trans‐fatty acids (TFAs) activated the MAPK pathway, leading to increased expression of phospholipase A2 (PLA2) and inflammatory mediators like COX‐2 and prostaglandin E2 [56]. The MAPK‐mediated regulation of PLA2 and other inflammatory cytokines could be relevant to the inflammation seen in HAPH. Chronic activation of endothelial MAPK has also been shown to disrupt haematopoiesis in the bone marrow by inducing an NF‐κB‐dependent inflammatory stress response. This inflammatory stress led to significant HSC dysfunction, suggesting that the MAPK‐NF‐κB axis plays a key role in promoting inflammation, which could affect the inflammatory microenvironment in HAPH [57].

### 4.4 MAPK and EndMT

The activation of MAPK pathways has been shown to play a crucial role in facilitating EndMT in HAPH by regulating key molecules like Snail and VCAM‐1. In atherosclerosis, the activation of the USF1‐NLRC5‐Smad2/3 axis facilitated EndMT, with MAPK‐related pathways playing a regulatory role [58]. This suggests that similar MAPK‐driven processes may contribute to EndMT in HAPH. In MDCK cells, the MAPK/Erk pathway was involved in regulating epithelial‐to‐mesenchymal transition (EMT), a process related to EndMT. Transcriptional factors like Snail were activated by the Erk/MAPK signalling pathway, indicating that MAPK can regulate genes related to mesenchymal transition [59]. The results indicate that the MAPK pathway is crucial in mediating EndMT, potentially contributing significantly to the development of HAPH.

## 5 BMP Signal Pathway

**FIGURE 4:**
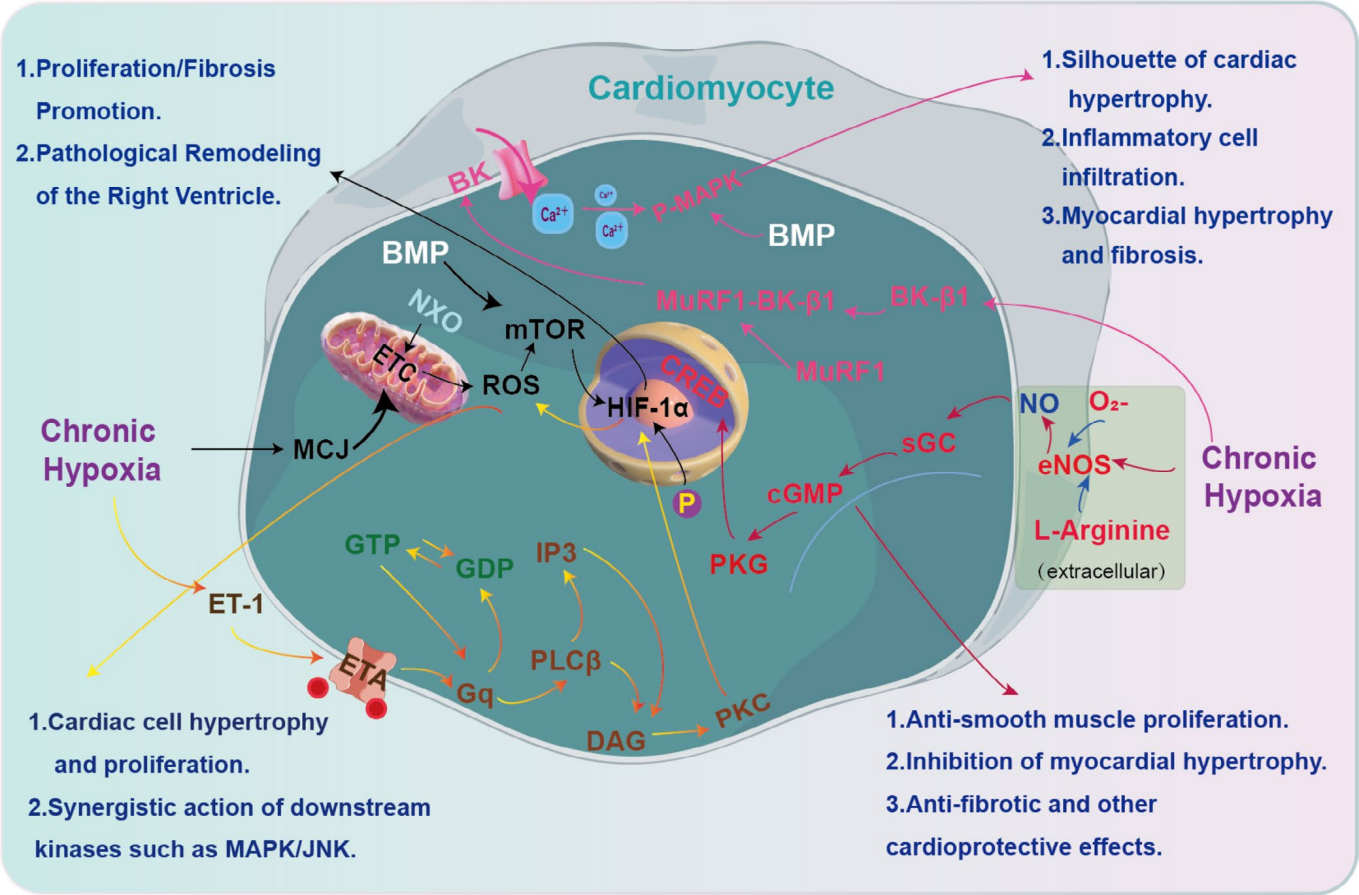
The bone morphogenetic protein (BMP) signalling pathway and its roles in development and disease. Ligand binding to a BMP receptor complex initiates intracellular SMAD‐dependent signalling to regulate gene expression. This pathway is critical for normal development, such as directing Muller glial cell differentiation in the postnatal retina. ETA receptor is a major cause of heritable pulmonary arterial hypertension, leading to aberrant signalling and increased cardiac cell hypertrophy and proliferation. BMP signalling is essential for tissue homeostasis and its dysregulation underlies HAPH pathologies.

BMP signalling pathway is a complex and essential regulatory system involved in various biological processes, such as development, tissue homeostasis and disease [60, 61]. As members of the transforming growth factor‐beta (TGF‐β) superfamily, BMPs have signalling pathways that are vital for numerous cellular processes. In the retina, a brief wave of BMP signalling is necessary for Müller glial differentiation. Between Post‐Natal Days 5 and 9, after neurogenesis has concluded, Smad1/5/8 signalling is transiently activated in the inner nuclear layer. Inhibiting BMP signalling at this time, whether in vitro or in vivo with BMP receptor antagonists or noggin, disrupts the expression of Müller glia‐specific genes like Rlbp1 and Glul. This leads to lasting retinal damage, including outer limiting membrane irregularities, rosette formation and diminished functional acuity [62]. BMPR2 functions as a major receptor in the BMP signalling pathway, associated with the TGF‐β receptor family and is essential for the development of embryos, vasculature and bones [63] (Figure 4). The BMPR2 gene in humans produces a protein consisting of 1038 amino acids, featuring extracellular, transmembrane, kinase and C‐terminal cytoplasmic regions. The binding of BMPs to a tetrameric assembly of BMPR2 and type 1 receptors starts signal transduction through SMAD proteins. This cascade regulates gene expression crucial for embryogenesis, organogenesis and bone development [63]. Researchers have identified over 300 BMPR2 mutations, with the majority being frameshift and nonsense types [64]. In BMPR2 mutation cases, oxidative injury is a frequent consequence. Studies show that mutations in the BMPR2 ligand‐binding domain, kinase domain and cytoplasmic tail lead to increased superoxide and peroxide production, as well as changes in oxidative stress‐related genes. Transgenic mice with mutations in the BMPR2 cytoplasmic tail exhibit elevated levels of isoprostanes and isofurans, indicating mitochondrial ROS generation. This oxidative stress is vascular‐specific in BMPR2 transgenic mouse lungs [64].

### 5.1 BMP Regulates Apoptosis and Cell Proliferation

BMPR2 dysfunction leads to increased endothelial cell apoptosis. For instance, BMPR2 mutations in HAPH patients increase endothelial cell susceptibility to apoptosis. Mutant BMPR2 expression in pulmonary endothelial cells promotes apoptosis and the release of factors that stimulate the proliferation of PASMCs. Overexpression of mutant BMPR2 (kinase‐deficient D485G mutation) in PAECs enhances apoptosis susceptibility and promotes PASMC proliferation [65]. Cytokines, such as TNF‐α and IL‐18, also exacerbate endothelial dysfunction in the context of BMPR2 dysfunction. In BMPR2‐silenced human lung microvascular endothelial cells (HLMVECs), TNF‐α induces P38‐MAPK activation and disrupts endothelial barrier function. This highlights the role of cytokines in promoting HAPH in the presence of impaired BMPR2 signalling [66]. As pulmonary hypertension progresses, BMPR2 signalling in pulmonary vascular endothelial cells shifts from promoting apoptosis in the early stages to driving a proliferative phenotype in later stages. MicroRNA‐125a has been identified as a regulator of this process, with its inhibition increasing endothelial proliferation and upregulating BMPR2 [67].

### 5.2 BMPR2 and ECM Remodelling

BMPR2 abnormalities are associated with ECM remodelling, a key feature of HAPH. BMPR2 deficiency enhances cellular responses to TGF‐β, leading to increased expression of ECM proteins such as fibrillin‐1 (FBN1) and integrins [68, 69, 70]. These changes contribute to the structural alterations in the pulmonary vasculature, exacerbating the disease.

### 5.3 The Role of BMPR2 in Sex Difference

Gender differences in PAH and HAPH are partly attributed to the BMPR2 signalling pathway. Studies show that oestrogen can downregulate BMPR2 expression through its receptor α (ERα), potentially explaining the higher prevalence of PAH in females [71], which is in contrast to the high incidence of HAPH in males. The deficiency of BMPR2 signalling results in increased proliferation and resistance to apoptosis in PASMCs and PAECs, promoting vascular remodelling and inflammation [72, 73]. Moreover, BMPR2 serves as a guardian for endothelial cells against heightened TGF‐β responses, which is frequently reduced in HAPH, resulting in additional vascular issues [74]. This suggests that sex‐specific differences in BMPR2 signalling contribute to the observed gender disparities in HAPH.

## 6 Emerging Signal Pathways

### 6.1 Wnt/β‐Catenin Signal Pathway in HAPH

**FIGURE 5:**
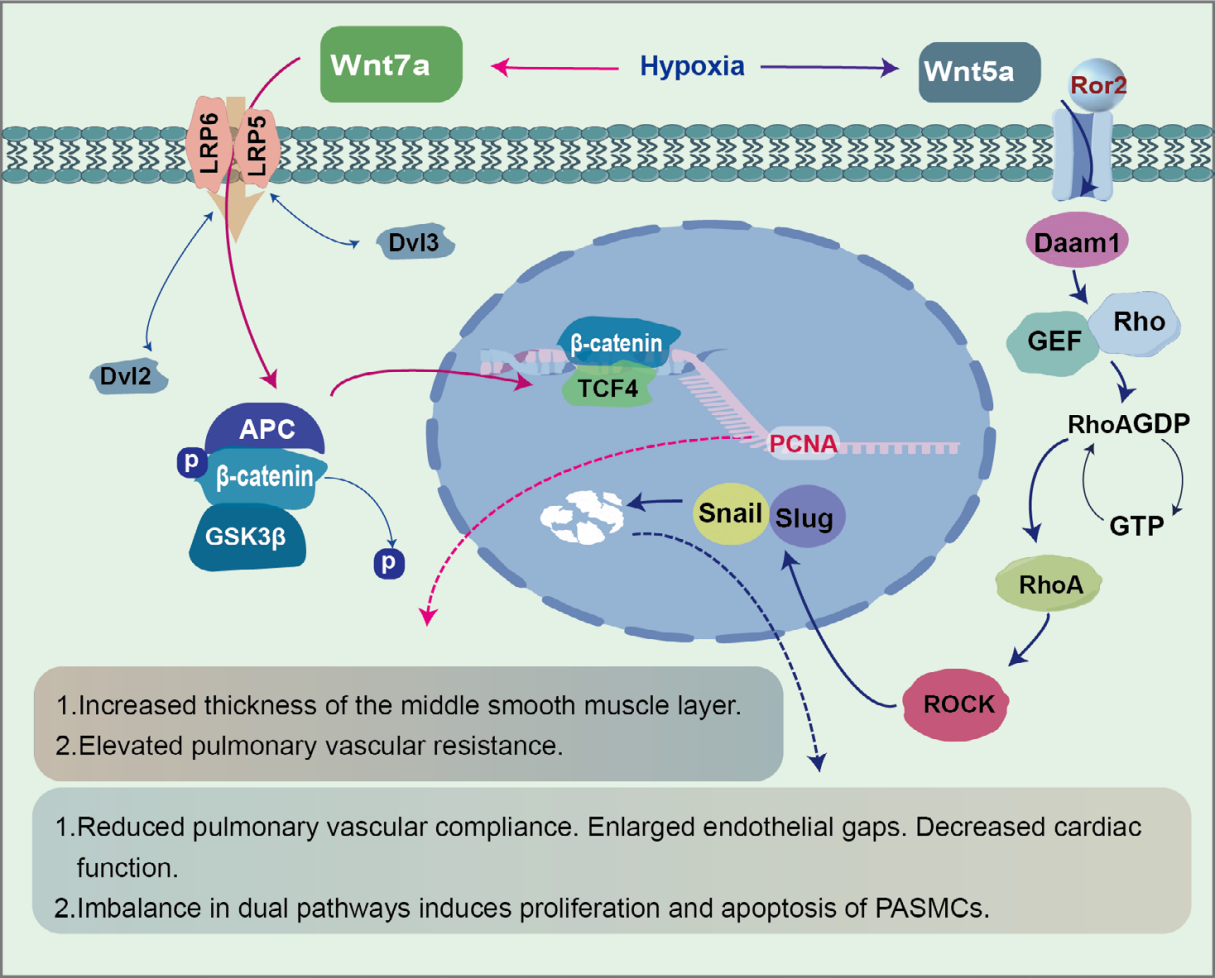
The role of Wnt/β‐catenin signalling in pulmonary vascular remodelling. The canonical Wnt pathway, when activated, stabilises β‐catenin and causes it to move into the nucleus, where it triggers the expression of genes important for cell cycle progression and extracellular matrix remodelling. In HAPH, this signalling cascade is hijacked, resulting in the uncontrolled growth and migration of PASMCs. Evidence from HPAH models shows that key pathway components (e.g., ROCK, Rho, PCNA) are upregulated and its inhibition can reverse vascular pathology.

The Wnt/β‐catenin pathway, known for its conservation, is crucial in several biological activities, including cell division, differentiation and tissue equilibrium [75, 76, 77]. In recent years, its role in the pathogenesis of pulmonary hypertension has gained significant attention. When myocardial injury occurs, the Wnt/β‐catenin pathway is triggered, leading to cardiac repair and regeneration through the stimulation of cardiomyocyte proliferation and differentiation [78, 79]. Dysregulation of this pathway in pulmonary hypertension causes PASMCs and endothelial cells to proliferate and migrate, which are important processes in the remodelling of pulmonary vessels. When the Wnt/β‐catenin pathway is activated, β‐catenin becomes stabilised, moves into the nucleus and triggers the transcription of genes that play a role in cell growth and extracellular matrix remodelling [80](Figure 5). Inhibition of this pathway with porcupine inhibitors, such as LGK‐974, in experimental models of pulmonary hypertension resulted in a significant improvement in cardiac hypertrophy and fibrosis. Genes critical to the pathway, including receptors (FZD4, FZD5), core elements (CTNNB1) and downstream targets (CCND1, VEGFA, AXIN2), also showed increased expression [81]. In animal studies, sulphur dioxide alleviates hypoxia‐induced pulmonary vascular remodelling in rats through the Dkk1/Wnt pathway [82]. In HAPH mice, NKD1 is downregulated and its overexpression inhibits hypoxia‐induced PASMC proliferation and migration by suppressing β‐catenin and ROS [83].

### 6.2 Notch Signal Pathway in HAPH

**FIGURE 6:**
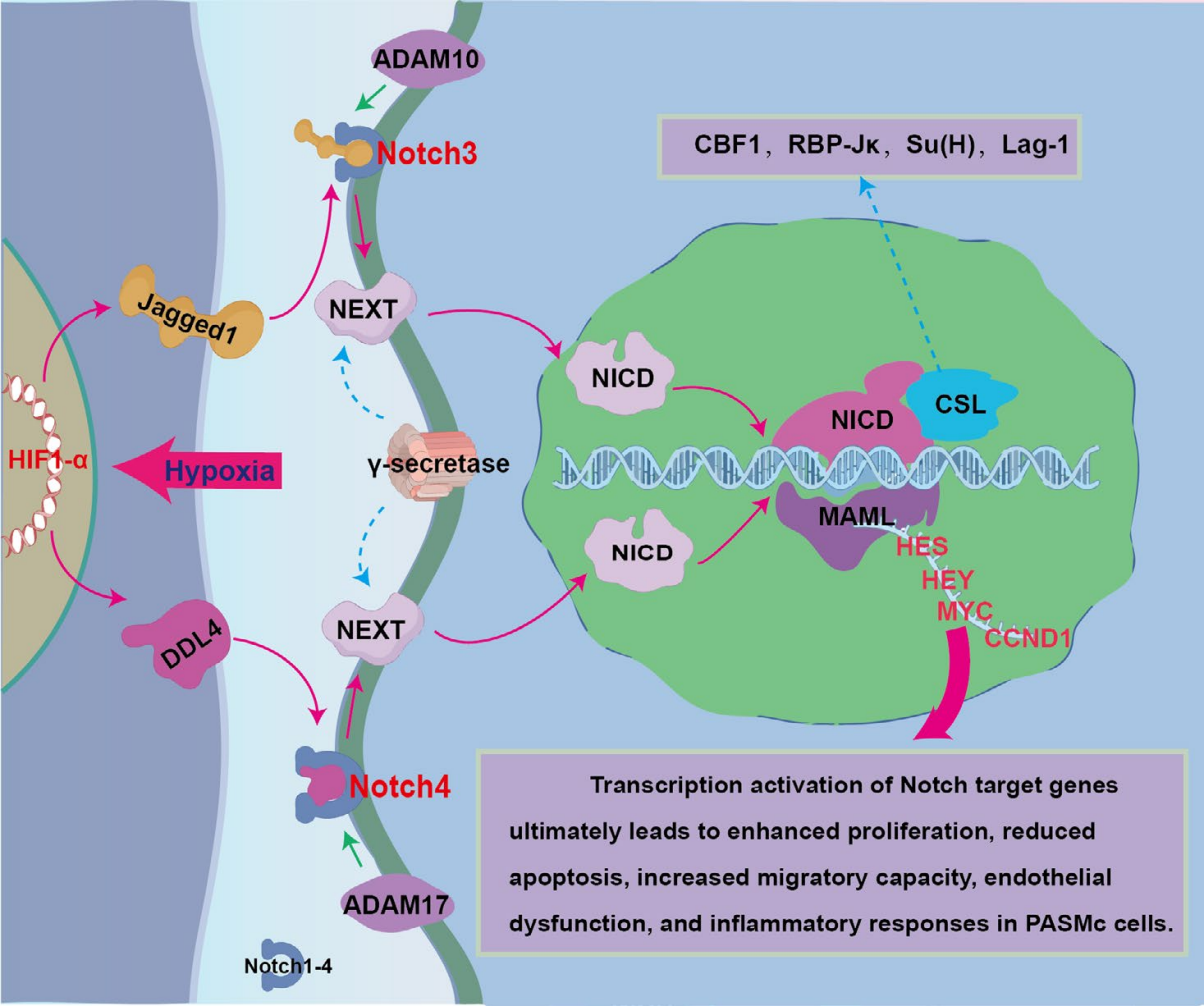
Notch signalling promotes vascular remodelling in HAPH through complex regulatory networks. The Notch pathway is activated in HAPH by hypoxia and regulatory axes such as HES, HEY, MYC and CCND1. Notch3 and Notch4 overexpression enhances PASMC proliferation via distinct mechanisms; this results in medial thickening and increased pulmonary vascular resistance. Targeted inhibition of Notch signalling presents a promising strategy for mitigating HAPH progression.

The Notch signalling pathway is a well‐preserved mechanism that controls embryonic development and physiological processes [84, 85] (Figure 6). In HAPH models, lncRNA Tug1 upregulates Foxc1 by sponging miR‐374c, activating Notch and promoting PASMC proliferation and vascular remodelling [86]. Hypoxia also increases VEGF/Notch signalling; miR‐203a‐3p suppresses VEGF‐A, reducing Notch1, VEGFR2 and Hes1, impairing endothelial angiogenesis [87]. Circ_0000790 similarly promotes PASMCs dysfunction via the miR‐374c/Foxc1 axis, implicating Notch in multi‐level regulation of HAPH [88]. Notch3 is overexpressed in pulmonary arteriolar smooth muscle cells and correlates with HAPH severity [89]. Genetic deletion or pharmacological inhibition of Notch3 prevents or reverses HAPH in mice, highlighting the critical role of the NOTCH3‐HES5 axis [89]. Mechanistically, Notch3 drives PASMC proliferation via Hes1‐dependent downregulation of p27Kip1 [90] and is activated by S1P and HIV Tat protein, indicating broad involvement across PAH subtypes including HAPH [91, 92]. Notch4 is upregulated in hypoxic pulmonary vessels and promotes medial thickening. In HAPH models and human PASMCs, hypoxia induces Notch4 and Delta‐4 expression. Notch4 activates ERK/JNK/P38 MAPK signalling, enhancing HPASMC proliferation and migration while inhibiting apoptosis. Silencing Notch4 attenuates vascular remodelling and hemodynamic changes, confirming its functional importance. Similar effects in lung cancer support its role in hypoxia‐driven cell behaviour [93, 94]. Targeting Notch4 may offer intervention potential in hypoxic vascular diseases.

### 6.3 Hippo‐YAP Signal Pathway in HAPH

**FIGURE 7:**
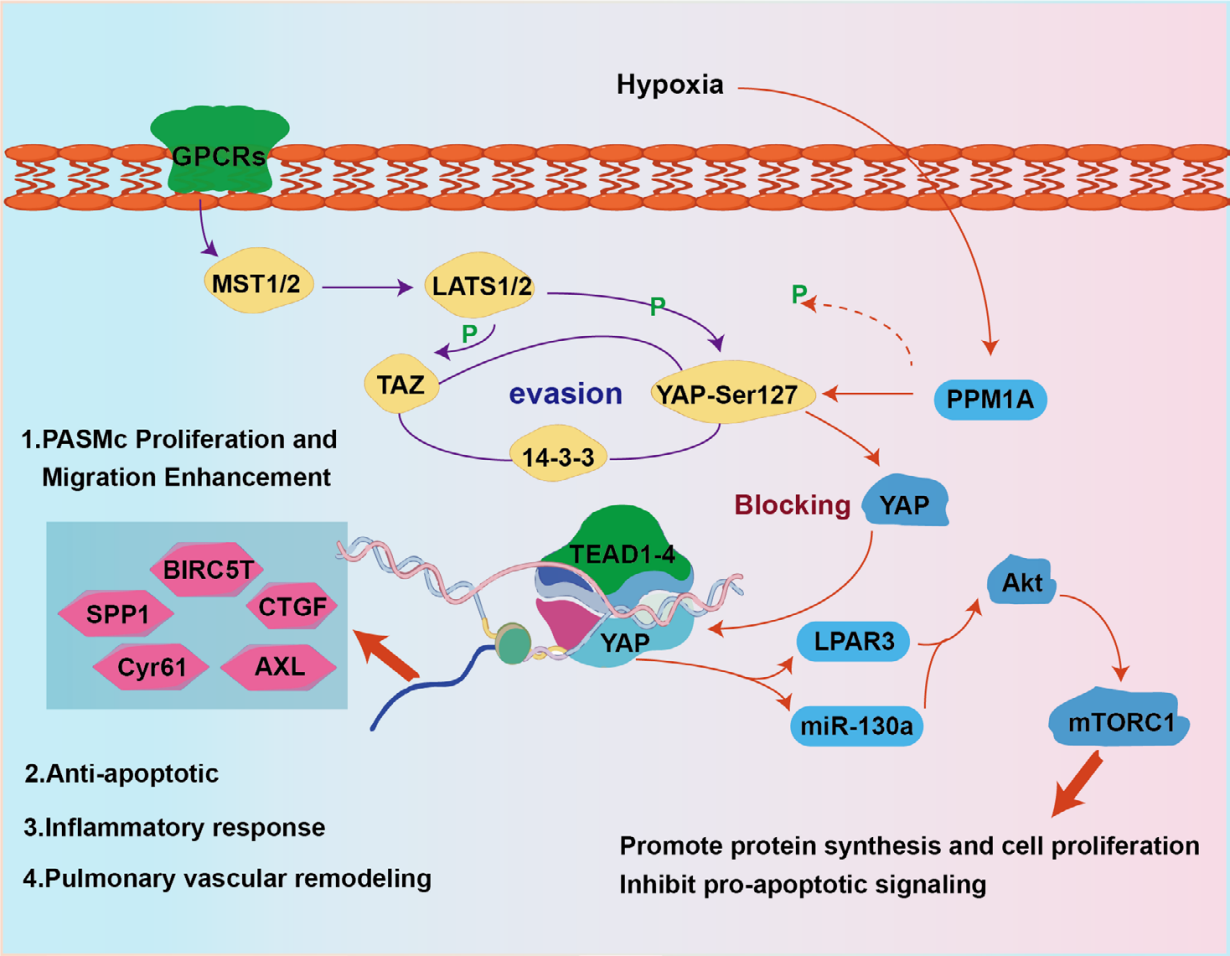
Central role of the dysregulated Hippo‐YAP pathway in the pathogenesis of HAPH. In HAPH, the Hippo pathway kinase LATS1/2 is inactivated, leading to the accumulation and nuclear translocation of the co‐activator YAP in pulmonary vascular cells. Activated YAP drives a pro‐remodelling programme by transcriptionally upregulating key factors like BIRC5T, SPP1, CTGF, AXL, Cyr61, while suppressing pro‐apoptotic signals. Inhibition of this axis reverses vascular remodelling in experimental models, highlighting its potential as a target.

The Hippo‐YAP signalling pathway is a highly conserved pathway that plays a crucial role in regulating organ size, cell proliferation and apoptosis [95]. In the context of HAPH, this pathway has emerged as a key regulator of pulmonary vascular remodelling and disease progression (Figure 7). In HAPH patients and animal models, LATS1 is inactivated and YAP is upregulated in PASMCs, driving their abnormal proliferation and survival [96]. This activation promotes mTOR–Akt signalling, increases HIF‐1α, Notch3 and β‐catenin levels, suppresses Bim expression and enhances cell proliferation. YAP also induces fibronectin secretion and ILK1 upregulation, which inhibits LATS1, forming a pathogenic feedback loop; targeting ILK1 with Cpd22 reverses vascular remodelling in models [97]. In HAPH, SIK1 downregulation enhances YAP activity, promoting PASMC proliferation and remodelling, effects associated with decreased YAP phosphorylation and enhanced movement into the nucleus [98]. Hypoxia‐driven YAP activation via LATS1/2 inhibition is central to HAPH, with similar mechanisms observed in cancers, supporting its broad role in hypoxic cell responses [99, 100]. These findings suggest that modulating the Hippo‐YAP pathway could be a valuable intervention strategy for HAPH, potentially reversing the maladaptive remodelling processes.

### 6.4 IL‐6 Dual Signal Pathways in HAPH

**FIGURE 8:**
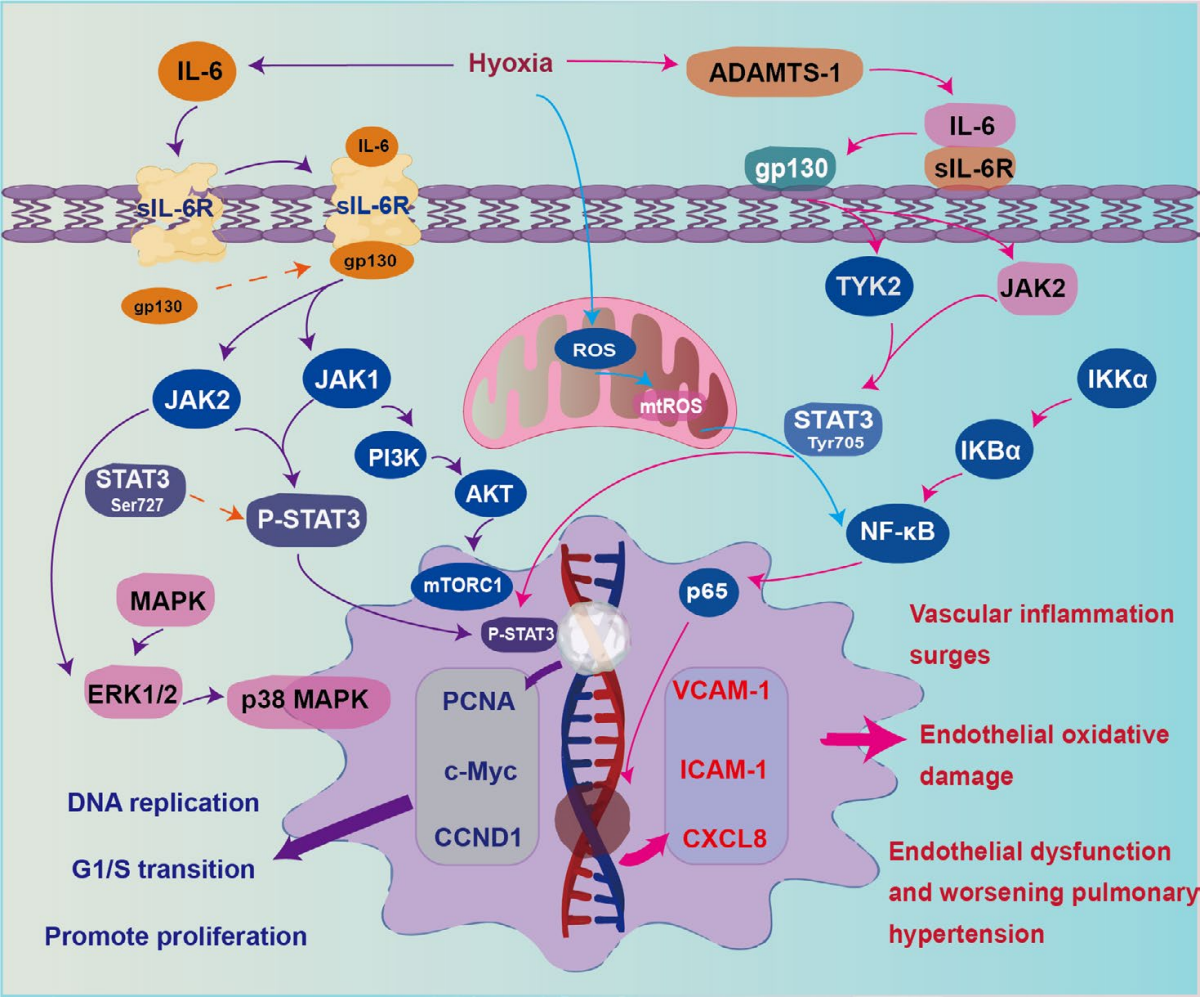
Cell‐specific IL‐6 signalling mechanisms in HAPH vascular remodelling. In HAPH, IL‐6 exerts distinct effects on different vascular cells. Classical signalling through the membrane IL‐6 receptor (sIL‐6R) on pulmonary artery smooth muscle cells (PASMCs) directly promotes hypertrophy and proliferation. Concurrently, IL‐6 trans‐signalling via the soluble IL‐6R (sIL‐6R) complex activates endothelial cells, triggering a pro‐inflammatory response. The convergence of these pathways leads to synergistic promotion of vascular inflammation, remodelling and the progression of HAPH.

IL‐6, known as Interleukin‐6, is a cytokine with a broad range of biological activities, operating through classical signalling (via membrane IL‐6R) and trans‐signalling (via soluble IL‐6R) [101, 102]. In HAPH, classical signalling may directly promote PASMC hypertrophy, while trans‐signalling activates endothelial cells, driving inflammation and vascular remodelling [103, 104](Figure 8). In HAPH models, senescent PASMCs secrete IL‐6 (SASP), promoting proliferation via mTOR/S6K1 activation, which is linked to IL‐6 release [105]. In HAPH patients and animal models, membrane IL‐6R is upregulated in PASMCs and IL‐6R antagonists were able to reverse experimental PH in HAPH rat models, suggesting that targeting IL‐6R could be a promising intervention strategy for HAPH [106, 107]. The IL‐6/sIL‐6R complex activates endothelial cells, inducing MCP‐1 and other inflammatory mediators via PI3K/AKT and MEK/ERK pathways, thereby amplifying vascular inflammation [104]. This trans‐signalling mechanism is conserved across diseases: it enhances endothelial proliferation in liver cancer via gp130/JAK2/STAT3 [108], induces MCP‐1 in brain endothelial cells [109] and promotes retinal endothelial dysfunction in diabetes, which can be mitigated by pathway inhibition [110]. Endothelial inflammation and vascular remodelling in HAPH are driven by IL‐6 trans‐signalling, suggesting that targeting this pathway could offer a new intervention strategy.

## 7 Epigenetic Regulation and HAPH

**FIGURE 9:**
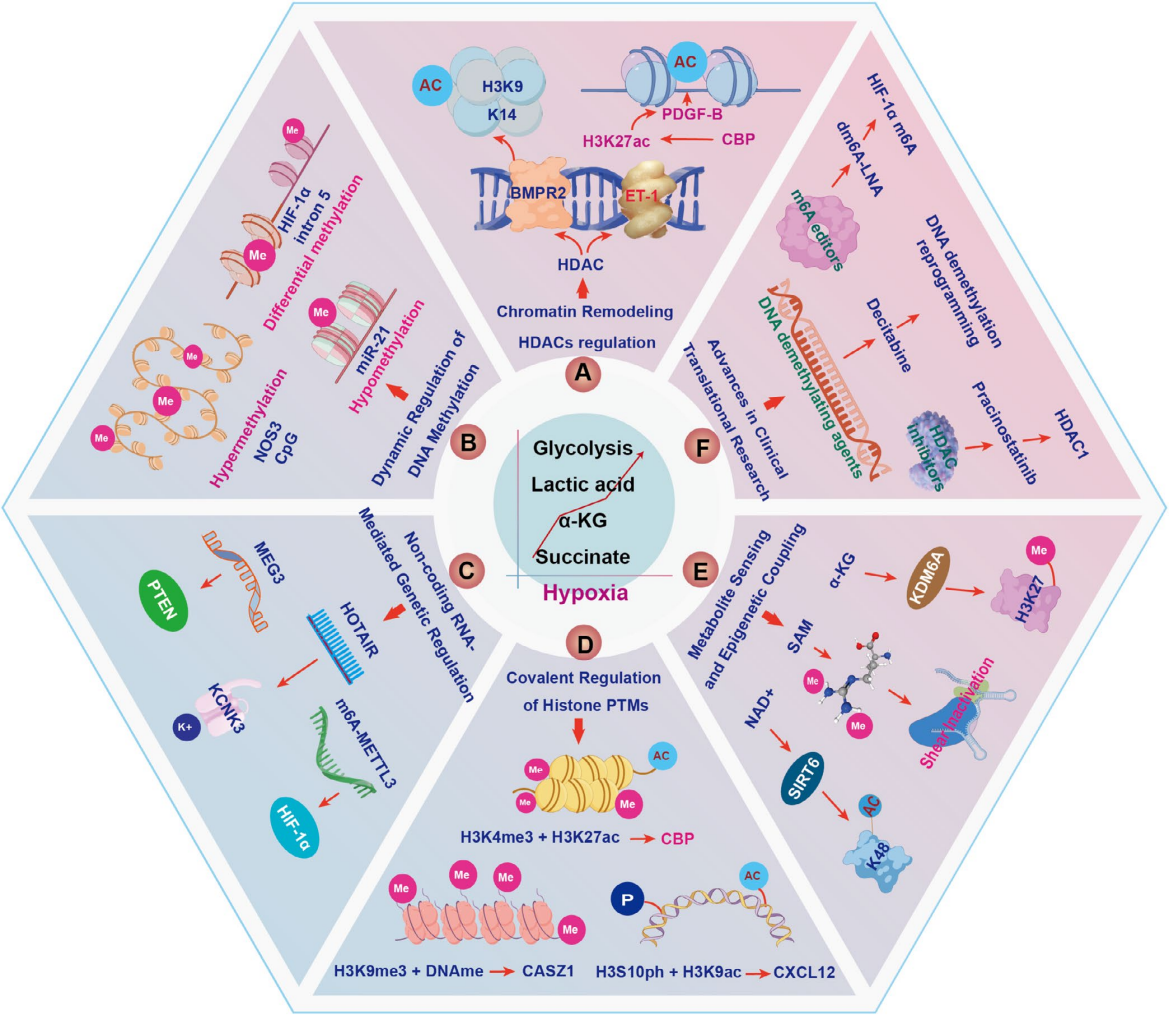
Multilayer epigenetic mechanisms driving vascular remodelling in HAPH. Epigenetic dysregulation, through histone modifications and non‐coding RNA networks, is a key driver of HAPH. These alterations transcriptionally suppress key genes like BMPR2 and post‐transcriptionally modulate pathways critical for vascular cell proliferation and inflammation. Hypoxia stabilises this maladaptive state by reinforcing a pro‐remodelling epigenetic landscape, often centred on enhanced HIF‐1α signalling. The dynamic nature of these changes highlights their potential as treatment targets.

The development of HAPH is significantly influenced by epigenetic mechanisms such as DNA methylation, histone changes and interference by non‐coding RNA. These modifications can alter gene expression without changing the DNA sequence, contributing to the development and progression of the disease [111](Figure 9). In HAPH, HDAC1/5 upregulation suppresses BMPR2. In HAPH models, HMGB1‐TLR4 signalling inhibits BMPR2, increasing plasma IL‐1β, IL‐6 and TNF‐α, upregulating vascular HMGB1/TLR4 and reducing p‐Smad1/5/8 and Id1 [112]. HMGB1 promotes PASMC proliferation and migration in vitro; HMGB1 or TLR4 inhibitors reverse these effects and restore BMPR2 signalling in vivo, improving hemodynamics and reducing remodelling. miR‐20a downregulates BMPR2; antagomiR‐20a enhances BMPR2 expression in hypoxic mice, attenuating vascular thickening and right ventricular hypertrophy and suppresses HPASMC proliferation [113]. circGSAP sequesters miR‐27a‐3p to upregulate BMPR2, protecting against PMEC dysfunction and remodelling [114]. circGSAP is reduced in HAPH and linked to poor prognosis; its overexpression improves survival and reduces pathology in HAPH rats [114]. Together, HDAC1/5‐mediated BMPR2 suppression is central to HAPH and targeting HMGB1/TLR4, miR‐20a or circGSAP may restore BMPR2 signalling for therapy. It is also reported that histone acetylation at promoter enhances HIF‐1α transcription, which upregulates genes involved in vascular remodelling, proliferation and inflammation. Hypoxia induces MLL1/p300 recruitment to the HOTAIR promoter, increasing H3K4me3 and acetylation to promote HOTAIR expression, which is dependent on HIF‐1α [115]. In pancreatic cancer, HIF‐1α induces miR‐646, which suppresses MIIP; MIIP in turn inhibits HDAC6, promoting HIF‐1α degradation, forming a negative feedback loop [116]. In liver ischemia/reperfusion, TGR5 knockout reduces SIRT3/FOXO3, enhancing HIF‐1α activity and injury severity [117]. Therefore, understanding epigenetic regulation may provide insight into HAPH mechanisms and potential interventions.

## 8 Intervention Strategies by Targeting Signal Pathway in HAPH

**FIGURE 10:**
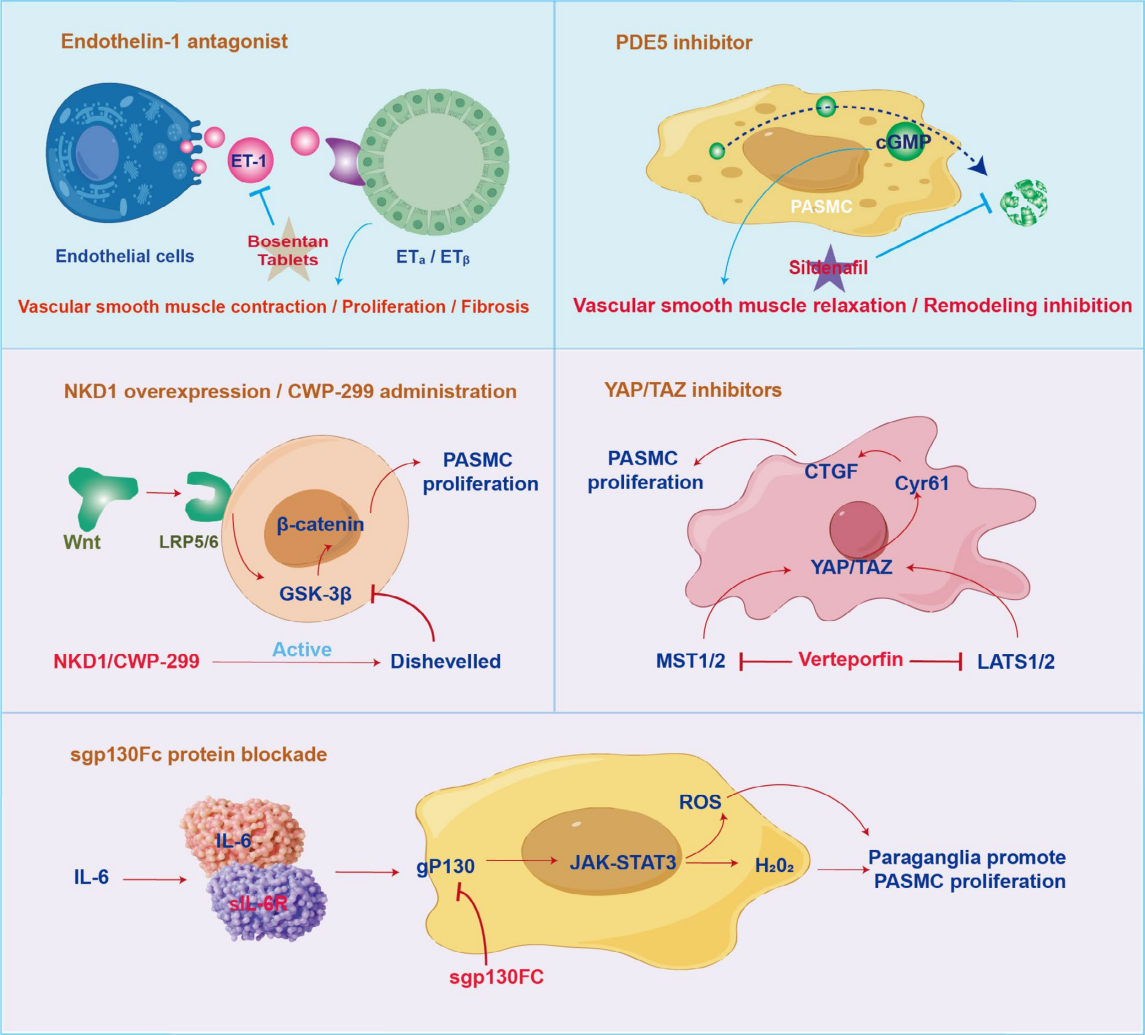
Key and growing research targets in the field of drug intervention for high altitude pulmonary hypertension.

Given the pivotal roles these signalling pathways play in the development and progression of HAPH, targeting them through inhibition or activation might be regarded as a crucial strategy for improving and alleviating HAPH (Figure 10). Intervention strategies targeting signalling pathways in HAPH can be prioritised based on translational potential and mechanistic specificity (Table 1). HIF signalling serves as a core upstream target, with HIF‐2α inhibitors (e.g., derivatives of PT2385) demonstrating efficacy in reversing hypoxia‐induced vascular remodelling by disrupting the ‘hypoxia‐pseudohypoxia’ loop [118, 119, 129]. BMP signalling rebalancing is another critical approach: novel pyrazolo[3,4‐b]pyridine derivatives like HLQ2g restore cGKI and BMP signalling [120], while ACTRIIA‐Fc (Sotatercept) inhibits PASMC proliferation by rebalancing the activin/GDF‐BMP axis [121]. Inflammatory pathways such as IL‐6 trans‐signalling can be targeted using sgp130Fc to mitigate remodelling without impairing classical regenerative functions [122, 123]. Downstream effectors offer additional intervention nodes. MAPK inhibitors like cyanidin‐3‐O‐β‐glucoside and MK2 inhibitors attenuate pulmonary hypertension via TGF‐β1/p38 MAPK/CREB or ERK/JNK/P38 pathways [124, 125]. Notch signalling can be blocked by γ‐secretase inhibitors (DAPT, RO4929097) to suppress NICD3‐HIF‐2α‐FoxM1‐mediated proliferation [126, 130]. Wnt/β‐catenin pathway inhibitors (LGK‐974, ETC‐159) targeting porcupine reduce fibrosis [127]. Additionally, Hippo‐YAP pathway modulators like Vetibopifen disrupt mechanotransduction‐driven remodelling [128]. These strategies collectively address the multilevel pathogenic network of HAPH, from upstream hypoxia sensing to downstream proliferative and fibrotic effectors.

**TABLE 1:** Therapeutic targets and interventions for HAPH.

Signalling pathway	Target molecule	Intervention agent	Mechanism of action	Translational potential	Preclinical/clinical evidence	References
HIF signalling	HIF‐2α	PT2385 derivatives	Disrupts hypoxia‐pseudohypoxia loop; reverses vascular remodelling	High (core upstream driver)	Reversed established HAPH in murine models; inhibits PASMC proliferation	[118, 119]
BMP signalling	BMPR2/cGKI	HLQ2g (pyrazolo[3,4‐b]pyridine derivative)	Restores cGKI and BMP signalling; attenuates PAH	High (pathway rebalancing)	Ameliorated hypoxic PH in rats via BMP signalling activation	[120]
	Activin/GDF‐BMP Axis	ACTRIIA‐Fc (Sotatercept)	Blocks activin/GDF signalling; rebalances BMP axis	High (Phase 3 trials for PAH)	Reduced pulmonary vascular resistance in preclinical models; clinical trials in Group 1 PAH	[121]
IL‐6 signalling	IL‐6 Trans‐signalling	sgp130Fc	Inhibits IL‐6 trans‐signalling; mitigates inflammation without impairing regenerative function	High (drug repurposing potential)	Improved survival and reduced remodelling in sepsis models; preclinical data in vascular disease	[122, 123]
MAPK signalling	p38 MAPK/CREB	Cyanidin‐3‐O‐β‐glucoside	Inhibits TGF‐β1/p38 MAPK/CREB pathway; reduces PASMC proliferation	Medium (downstream effector)	Attenuated MCT‐induced PAH in rats via MAPK suppression	[124]
	MK2	MK2 Inhibitor	Blocks ERK/JNK/P38 MAPK cascades; ameliorates pulmonary hypertension	Medium (preclinical validation)	Protective in experimental PH models via MAPK pathway inhibition	[125]
Notch signalling	Notch3/HIF‐2α/FoxM1	γ‐Secretase Inhibitors (DAPT, RO4929097)	Suppresses NICD3‐HIF‐2α‐FoxM1‐driven proliferation; inhibits PASMC migration	Medium (targeted pathway)	Prevented ductus arteriosus smooth muscle cell proliferation in vitro	[126]
Wnt/β‐catenin signalling	Porcupine (Wnt secretion)	LGK‐974, ETC‐159	Inhibits Wnt ligand secretion; reduces vascular fibrosis	Medium (emerging target)	Inhibited fibrosis in preclinical models via Wnt/β‐catenin suppression	[127]
Hippo‐YAP signalling	YAP/TAZ‐TEAD	Vetibopifen	Disrupts mechanotransduction‐driven remodelling; inhibits YAP nuclear translocation	Low (early‐stage development)	Blocked YAP‐mediated proliferation in cancer models; theoretical relevance to HAPH fibrosis	[128]

Despite promising preclinical data, translational barriers exist. Safety is a primary concern: systemic HIF‐2α inhibition carries a risk of anaemia due to suppressed erythropoietin, which may be detrimental in high‐altitude residents relying on compensatory polycythemia [131]. Similarly, while IL‐6 and Notch inhibitors are effective, they pose risks of infection and gastrointestinal toxicity, respectively [132, 133]. Furthermore, most current clinical trials focus on Group 1 PAH; there is an urgent need for dedicated clinical studies involving distinct cohorts of patients with HAPH to validate these targets specifically in the context of chronic hypoxic exposure.

## 9 The Integration of Classical and Emerging Pathways

**FIGURE 11:**
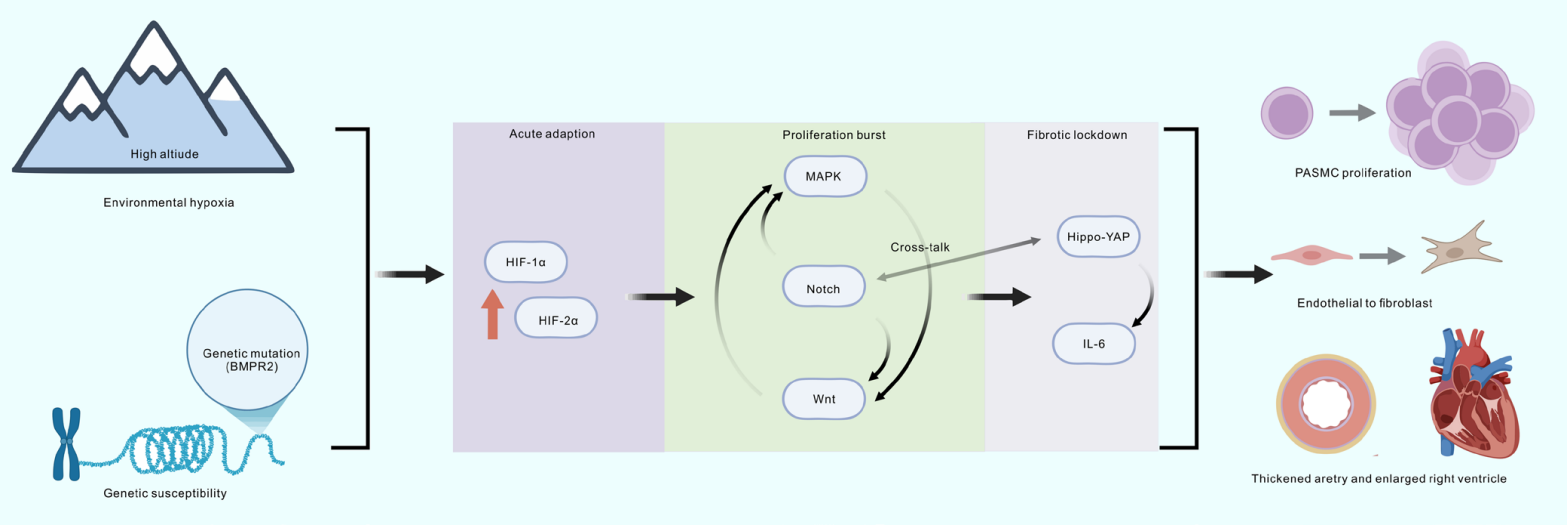
Schematic illustration of hypoxia‐induced phased signalling reprogramming model. This model elucidates how continuous hypobaric hypoxia reprogrammes pulmonary vascular cell fate through three coordinated dimensions: (i) the interaction initiation: environmental hypoxia intersects with genetic susceptibility to lower the threshold for pathological signalling. (ii) The signalling network: classical pathways trigger the activation of emerging regulators. (iii) The temporal handover: the transition from acute metabolic adaptation to maladaptive proliferation and finally to irreversible fibrosis, ultimately determining the cell fate of vascular remodelling.

Although detailed individually, current evidence suggests that HAPH pathogenesis relies on the functional synergy between classical and emerging pathways. Acting as an environmental trigger, studies have shown that HIF‐1α further promotes vascular remodelling by activating the emerging Hippo‐YAP pathway [134]. Additionally, the interaction between the HIF pathway and the MAPK pathway is considered one of the key mechanisms in the pathophysiology of HAPH [135]. This suggests that emerging pathways often require ‘priming’ by classic hypoxic sensors to initiate pathological signalling. Interactions between pathways can also manifest as the loss of inhibitory control. In the BMP signalling pathway, mutations in the BMPR2 gene are closely associated with the development of PAH. Under normal conditions, BMP maintains homeostasis; however, dysregulation of BMP signalling not only affects endothelial cell function but also promotes the proliferation of vascular smooth muscle cells through interactions with the Notch signalling pathway [136, 137]. Additionally, the interaction between BMP and Wnt signalling plays a significant role, particularly in the proliferation and survival of vascular endothelial cells [138]. As a critical inflammatory mediator, IL‐6 plays a pivotal role through its classical signalling pathway [139, 140]. Furthermore, the interaction between IL‐6 and other inflammatory pathways, such as NF‐κB, is considered a key factor in the pathological process [141]. This inflammatory network often operates in parallel with the Notch4‐MAPK axis described earlier [93], creating a feed‐forward loop that sustains vascular inflammation. Ultimately, these diverse interactions reach a consensus on cell fate. The proliferative signals from Notch and Wnt converge to upregulate Cyclin D1 [81], while MAPK and Wnt synergistically promote EndMT via the transcription factor Snail [59]. The signalling landscape of HAPH shifts dynamically across three overlapping phases. We have clarified the specific temporal roles of these pathways as follows: (i) Early phase: adaptive response and acute vasoconstriction. In the acute phase, the response is primarily functional rather than structural. Hypoxia rapidly stabilises HIF‐1α, which regulates ion channels and upregulates vasoconstrictors like Endothelin‐1 and VEGF. This is initially an adaptive mechanism to optimise ventilation/perfusion matching. At this stage, vascular cells have not yet undergone significant proliferation and the changes are largely reversible upon re‐oxygenation. (ii) Intermediate phase: maladaptive transition and cellular proliferation. As hypoxia persists, the sustained upregulation of downstream targets (VEGF from the HIF pathway) triggers secondary proliferation pathways. Notch3 is activated and contributes to the transition of smooth muscle cells from a contractile to a synthetic phenotype. Notch4 activates the MAPK cascade (ERK/JNK/P38) to drive uncontrolled PASMC proliferation. Wnt/β‐catenin signalling begins to upregulate Cyclin D1, pushing cells past cell cycle checkpoints. The initially protective vasoconstriction transforms into fixed structural remodelling. (iii) Late phase: adverse remodelling, fibrosis and decompensation. In the chronic stage, mechanical stiffness and sustained inflammation become self‐perpetuating drivers. Sensing increased matrix stiffness (mechanotransduction), YAP nuclear translocation promotes collagen deposition and fibrosis, locking the vessel wall in a rigid state. Chronic inflammation mediated by IL‐6 amplifies the remodelling process and contributes directly to right ventricular fibrosis and failure. The continued suppression of BMP signalling exacerbates endothelial dysfunction and adventitial fibrosis. The outcome is irreversible vascular fibrosis and right heart failure. The temporal sequence explains why late‐stage HAPH is resistant to therapy. Early activation of HIF creates a ‘permissive environment’ (metabolic reprogramming). Subsequent activation of MAPK and Notch drives the ‘proliferative burst’. Finally, the activation of mechanosensitive pathways like Hippo‐YAP and inflammatory loops (IL‐6) results in ‘fibrotic lockdown’. Here, we propose a unified model where HAPH pathogenesis is not a linear event but a coordinated network reprogramming process. Hypoxia acts as the initiator, genetic susceptibility provides the substrate and the signal network executes the cell fate decision through a temporal handover mechanism (Figure 11). In conclusion, the multi‐level interactions between classical and emerging signalling pathways collectively influence the development of HAPH. These pathway interactions not only elucidate the complex pathological mechanisms but also provide new directions for future therapeutic strategies.

## 10 Conclusions and Perspectives

HAPH is a complex and multifaceted disease that arises from the interplay of genetic predisposition, environmental hypoxia and dysregulated cellular signalling networks. In conclusion, HAPH is driven by a highly interconnected signalling network where classical pathways and emerging regulators synergize to determine cell fate. Rather than acting linearly, these pathways converge on shared downstream effectors to reach a pathological consensus. Each of these pathways contributes to the progression of HAPH through distinct mechanisms, including acute hypoxic vasoconstriction, chronic vascular remodelling and right ventricular decompensation. Inhibiting the HIF signalling pathway, particularly, HIF‐2α, has been shown to delay the onset and progression of HAPH. Similarly, modulating the MAPK pathway through agents like 17β‐estradiol can reduce vascular proliferation and inflammation. Inhibitors of the BMP pathway, such as DMH1 and the Wnt/β‐catenin pathway, like LGK‐974, have demonstrated efficacy in reducing fibrosis and improving cardiac function. Additionally, targeting the Notch, Hippo‐YAP and IL‐6 pathways holds potential for alleviating vascular remodelling and right ventricular dysfunction. Future research in HAPH should focus on several key areas to advance our understanding and treatment of this disease:

1. *Molecular mechanisms*: Further elucidate the molecular mechanisms underlying the activation and crosstalk of signalling pathways in HAPH. This includes exploring the role of epigenetic modifications, non‐coding RNAs and post‐translational modifications in regulating these pathways.
2. *Genetic and environmental interactions*: Investigate the complex interactions between genetic predisposition and environmental factors in HAPH. This involves identifying novel genetic variants and understanding how they modulate hypoxia tolerance and disease susceptibility.
3. *Intervention target validation*: Conduct preclinical and clinical studies to validate the efficacy and safety of intervention agents targeting key signalling pathways. This includes developing and testing small molecules, biologics and gene therapies that can modulate HIF, MAPK, BMP, Wnt/β‐catenin, Notch, Hippo‐YAP and IL‐6 signalling.
4. *Biomarker development*: Identify and validate biomarkers that can predict disease progression, monitor treatment response and stratify patients for personalised therapy. This involves exploring circulating biomarkers, imaging modalities and omics‐based approaches to develop comprehensive diagnostic and prognostic tools.
5. *Systems biology approaches*: Utilise systems biology and computational modelling to integrate multi‐omics data and generate a holistic understanding of the signalling networks in HAPH. This can help identify novel intervention targets and optimise combination therapies.
6. *Clinical trials*: Design and conduct well‐powered clinical trials to evaluate the effectiveness of targeted therapies in diverse patient populations. This includes exploring the potential of combination therapies that target multiple pathways simultaneously to achieve synergistic effects.

HAPH remains a significant public health challenge, particularly in high‐altitude regions. Understanding the intricate signalling networks that drive its pathogenesis is crucial for developing effective intervention strategies. By targeting key signalling pathways and leveraging advances in molecular biology and systems medicine, we can pave the way for novel treatments that can reverse the maladaptive remodelling processes and improve outcomes for patients with HAPH. Future research must continue to explore the complex interplay between genetics, environment and signalling pathways to unlock new intervention avenues and ultimately conquer this debilitating disease.

## Author Contributions

**Sheng Ding:** conceptualisation, writing – original draft, writing – review and editing. **Ju Chen:** conceptualisation, writing – original draft, writing – review and editing. **Zhaoyang Li:** writing – original draft. **Yang Yu:** writing – original draft. **Weijie Wang:** writing – original draft. **Yan Liao:** writing – original draft. **Jin Yang:** resources, supervision. **Dianxiang Lu:** supervision, writing – review and editing. **Yujiang Fan:** writing – review and editing.

## Funding

This work was supported by the Natural Science Foundation of Sichuan Province (2024YFFK0280 and 2024ZDZX0010), National Natural Science Foundation of China (82402725 and 82374148), Chengdu University and Natural Science Foundation of Tibet Autonomous Region.

## Ethics Statement

The authors have nothing to report.

## Conflicts of Interest

The authors declare no conflicts of interest.

## Data Availability

Data sharing not applicable to this article as no datasets were generated or analysed during the current study.
